# Reducing Decision to Incision Time Interval for Emergency Cesarean Sections: 24 Months’ Experience from Rural Sierra Leone

**DOI:** 10.3390/ijerph18168581

**Published:** 2021-08-13

**Authors:** Lahai Tucker, Anna Frühauf, Isata Dumbuya, Paul Muwanguzi, Marta Lado, Daniel Lavallie, Mohamed Sheku, Chiyembekezo Kachimanga

**Affiliations:** 1 Partners In Health Sierra Leone, Koidu Town, Kono District, Sierra Leone; ltucker@pih.org (L.T.); afruehauf@pih.org (A.F.); idumbuya@pih.org (I.D.); pmuwanguzi@pih.org (P.M.); mlado@pih.org (M.L.); 2 Ministry of Health and Sanitation, Koidu Government Hospital, Koidu Town, Kono District, Sierra Leone; danlavalie1023@yahoo.com; 3 Ministry of Health and Sanitation, Makeni Regional Hospital, Makeni City, Bombali District, Sierra Leone; mgsheku42@gmail.com; 4 Partners in Health Malawi, Neno 313100, Malawi

**Keywords:** Sierra Leone, cesarean delivery, quality of obstetric care, audit and feedback, decision to incision time interval

## Abstract

Background: This study aimed at describing the changes in the completeness of documentation and changes in decision to incision time interval of emergency cesarean sections after an audit and feedback project a rural hospital in Sierra Leone. Methods: We documented and monitored the decision and incision times for emergency cesarean sections over the course of two years. Year one focused on the introduction of the project and year two focused on the continuous monitoring of the project. We compared the completeness of decision to incision data and used the 30-min benchmark as target for the decision to incision time interval. Results: A total of 762 emergency cesarean sections were included. While the completion of decision time data (72%) did not change between the two reporting periods, documentation of incision time increased from 95% to 98% (*p* < 0.001). Complete documentation for both decision and incision time was available for 540 (70.9%) emergency cesarean sections. The decision to incision time interval decreased from 105 min to 42 min (*p* < 0.001). The proportion of cesarean sections started within 30 min increased from 8.5% to 37% (*p* < 0.001). Conclusion: Although not all cesarean sections were performed within the 30-min threshold, the decision to incision interval decreased significantly. Improvements in documentation and routine reporting of the decision to incision time interval is recommended.

## 1. Introduction

Sierra Leone reports one of the highest rates of maternal and neonatal mortality and morbidity worldwide. In 2013, a maternal mortality ratio of 1165 deaths per 100,000 live births and neonatal mortality rate of 39 deaths per 1000 live births were recorded [1]. Poor maternal and neonatal outcomes were exacerbated by the Ebola virus disease (EVD) outbreak between 2014 and 2016, which devastated an already fragile health system [2,3,4]. To address the high maternal and neonatal mortality, access to comprehensive emergency obstetric and neonatal care, including cesarean sections, was prioritized by the Ministry of Health and Sanitation (MoHS) of Sierra Leone [5].

Cesarean section is the most common surgical procedure performed in Sierra Leone, estimated at 23% of all facility births in 2016 [6]. Although cesarean section is life-saving to women and neonates, it is unfortunately associated with high mortality in Sierra Leone [6,7]. In 2016, the cesarean section mortality rate was 1.5%, which is 30 times higher than the mortality rate in high income countries [8]. ⁠Perinatal mortality after cesarean section is also alarmingly high at 190 per 1000 births [9]. For example, in a study of 1274 cesarean sections and 1376 babies that were delivered, 11.3% and 3.8% of the babies were fresh still births and macerated still births, respectively. Among the live births, early neonatal deaths occurred in 4.5% of the babies [9]. Therefore, improving the quality and safety of the cesarean section is essential to improve maternal and neonatal outcomes in Sierra Leone.

Routinely measuring the quality of cesarean sections is fundamental in assessing health worker performance and the overall quality of surgical procedures, in turn, positively impacting neonatal and maternal mortality and morbidity [10]. The decision to incision time interval for emergency cesarean sections has been proposed as a key indicator for assessing the quality of cesarean sections [11].

The American College of Obstetrics and Gynecology recommends that the decision to incision time interval for emergency cesarean section should not exceed 30 min, and the 30 min benchmark has been used in many different settings to assess and improve the performance of obstetric teams responsible for responding to emergencies [12,13,14,15,16].

Despite the high cesarean section mortality rate in Sierra Leone, the quality of cesarean sections is rarely assessed and the decision and incision time interval is not one of the routinely collected indicators in Sierra Leone. As common in other resource-limited settings, there is no set benchmark for responding to emergency cesarean sections and the decision to incision time interval is rarely recorded [17].

Audits and feedback are ways of improving the performance of health care workers against an agreed standard [18,19]. Through the employment of this quality improvement method, health care professionals are incentivized to improve their performance, and consequently, care provision and patient outcomes, by regularly reviewing available data [20].

Immediately after the EVD epidemic in Sierra Leone, there was an acute need to improve the quality of maternal and neonatal services at Koidu Government Hospital (KGH). KGH was the only secondary hospital in the Kono District, a rural eastern district with a population of over 500,000 people [21]. Even though the hospital served as the only facility offering comprehensive emergency obstetric and neonatal care (CEmONC), KGH’s obstetric unit was supported by only 5 nurse-midwives, 15 nurses, and 5 nursing aides (who were not formally trained in midwifery), almost 50% of whom were unpaid volunteers. Cesarean sections were performed by two general medical doctors and one associate doctor, all of which were officially stationed in other departments. The hospital had no obstetrician and one nurse-anesthetist worked in both the maternity and general surgery theater. The facility conducted over 70 facility births monthly, of which 40–50% were cesarean sections.

Partners In Health, a non-governmental organization, started supporting the MoHS at KGH in late 2015. The organization addressed challenges across the World Health Organization building blocks, including supporting human resource gaps, providing necessary equipment and supplies, making changes in the infrastructure, and developing quality improvement initiatives in the obstetric unit. Some of the initiatives implemented from 2016–2017 in the obstetric unit are summarized in Supplementary Table S1.

One challenge identified was the high decision to incision time interval for cesarean sections, with most surgical procedures not meeting the 30-min benchmark. Between October and November 2016, the average decision to incision time interval was 262 min. Secondly, the documentation of decision time and incision time was not universal and there was no register documenting both decision time and incision time. Both times were recorded in separate files stored at different places, with the decision time being recorded on inpatient charts, while the incision time was recorded in the cesarean section register. To improve quality of documentation and reduce the decision to incision time interval, an audit and feedback on monitoring and measuring of the decision to incision time interval was introduced towards the end of 2016. The project aimed at using rigorous documentation of time intervals to collectively identify, discuss, and tackle delays in obstetric care, while continuing to use the data as an on-going feedback tool allowing for further adaptation. The overall objective of the initiative was to reduce the decision to incision time interval at the hospital to below the 30-min benchmark.

This report, therefore, (a) assesses the completeness of decision to incision time interval documentation at a rural secondary hospital in Sierra Leone, and (b) measures the changes in decision to incision time interval for emergency cesareans sections over the course of two one-year time periods, with one year focused on the introduction of the project, and one year focused on the continuous monitoring of the project.

## 2. Materials and Methods

A retrospective review of the decision to incision time interval register for emergency cesarean sections at KGH in the Kono district, Sierra Leone was conducted. The data were reviewed for 24 months, between October 2016 and September 2018 [21].

At the start of audit and feedback project, all health care workers in the obstetric unit, including theatre staff, were sensitized on the importance of documenting decision time and incision time and reducing the decision to incision time interval in October 2016. These changes were introduced within the first 6 months (October 2016 to March 2017), and were agreed on by the all health care workers in the obstetric unit. Firstly, all obstetric unit staff were trained on documentation and data quality. Secondly, the senior midwives working in the obstetric unit were officially assigned with the responsibility of transferring decision time data from patient charts to an existing cesarean section log book for all cesarean sections. These midwives were generally regarded as leaders in the unit, and they were made accountable if there were challenges with documentation. The senior midwives were also responsible for continuous mentorship to the rest of the nurses and other members of the obstetric staff. Lastly, a dedicated monitoring and evaluation (M&E) staff member was put in charge of aggregating the decision to incision time interval into an electronic decision to incision time interval register on a monthly basis.

Every month, one nurse-midwife (nurse midwife hired by PIH) in the obstetric unit reviewed the data together with the M&E staff to flag challenges in the documentation of the decision and incision time interval and to calculate the average decision and incision time. This was followed by a monthly obstetric team meeting where feedback was provided to the entire obstetric team, challenges identified were discussed, and solutions to address these were collectively developed. Where necessary, meetings with individual team members were also organized. The senior nurse-midwives were responsible for following up on the action points. Other meetings, like the MoHS mandatory monthly maternal death surveillance review meetings, provided other avenues of discussing the decision to incision time interval data findings.

After six months (March 2017), preliminary data showed a decision to incision interval of 222 min. These data were presented to the KGH hospital administration (composed of a medical superintendent, matron, and hospital secretary) and the obstetric unit team, and reducing the decision to incision time interval was made a priority. The decision to incision time interval indicator was added to the list of routine hospital indicators. Finally, it was agreed that this indicator will continue to be reviewed monthly at the obstetric unit meetings and quarterly at a larger hospital wide meeting.

**Figure 1:**
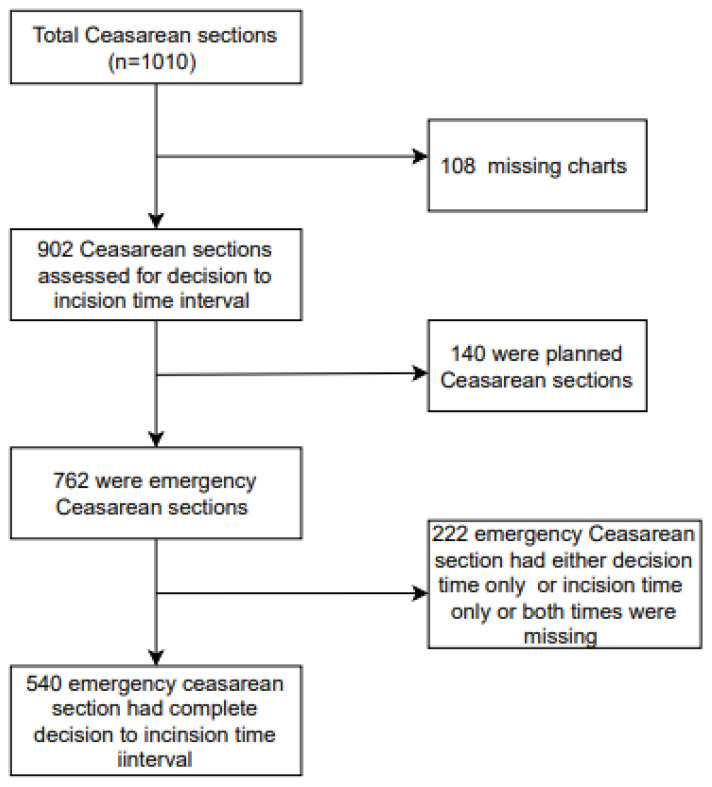
Cesarean sections at Koidu Government Hospital between October 2016 and September 2018.

In this study, all women giving birth by emergency cesarean section and who were recorded in the decision to incision interval register at KGH were included in the analysis. At the time, cesarean section was either classified as emergency or planned. Additionally, locally classified indications of cesarean section were used, as was the practice in other health facilities in the country (Supplementary Table S2) [7]. All other cesarean sections that were classified as planned and those missing patients’ charts were not included in the study (Figure 1).

The following outcomes in the electronic decision to incision interval register were extracted and measured:

(1) Completeness of documentation of decision time for surgery (in hours and minutes) and incision time (in hours and minutes). The decision time was the time when the clinician decided to perform emergency cesarean section. The incision time was the time when the clinician made the first incision to start the surgery. If one or both of the times were missing or incomplete, the decision time or incision time documentation was regarded as incomplete.
(2) Decision to incision time interval (in minutes). We converted the decision time and incision time to minutes and calculated the difference as the decision to incision time interval. The decision to incision time interval was further categorized to: within 30 min, 31–75 min, or over 75 min.
(3) Indications for cesarean sections. We used the locally defined indications and only included indications that were categorized as emergency cesarean sections (Supplementary Table S2).
(4) The time of the day, categorized into day (when the decision time was between 8 a.m. and 5 p.m.) or night (when the decision time was between 5 p.m. and 8 a.m.).
(5) The day of the week, categorized as weekday (Monday to Friday) or weekend (Saturday and Sunday).
(6) Maternal outcomes (alive or death) and neonatal outcomes (alive, fresh still birth, or neonatal death). As the study aimed at improving the ability of health care workers to respond to emergency cesarean section using the audit and feedback, these were the outcomes that were chosen to be tracked by the obstetric unit.

We compared the outcomes between two periods: period 1, one year time between October 2016 to September 2017, and period 2, one year time between October 2017 to September 2018. Period 1 was the initial period when the decision to incision time interval project were first implemented and rapid changes were made to improve the quality of services in the KGH obstetric unit (Supplementary Table S1). During period 2, initial health system strengthening support, including the decision to incision time interval project, was maintained and monitored.

All data from the decision to incision electronic register were exported to STATA version 15 for cleaning and analysis. Descriptive statistics were used to describe all the variables. Comparison between period 1 and period 2 for categorical variables was done using the Chi-squared test and Fisher’s exact test based on expected frequency, and the Mann–Whitney U test was performed for continuous variables. We used *p* < 0.05 as a cut-off for statistical significance.

The study was approved by the Office of Sierra Leone Ethics and Scientific Review Committee. As the study used routinely collected data and the data were analyzed retrospectively, we did not obtain informed consent.

## 3. Results

### 3.1. Emergency Cesarean Sections at Koidu Government Hospital

Between October 2016 and September 2018, 1010 cesarean sections were performed at KGH, representing 41% of all births at the facility (Figure 1). After introducing an audit and feedback project on the decision to incision time interval in mid-October 2016, 902 (89%) cesarean sections were captured in the register. One-hundred eight cesarean sections were not entered in the register, mainly because their files were missing. Among the 902 cesarean sections, 84.5% (*n* = 762) were emergency cesarean sections. Emergency cesarean sections performed increased from 40% (*n* = 305) to 60% (*n* = 457) between period 1 and 2.

### 3.2. Documentation of Decision to Incision Time Interval for Emergency Cesarean Sections

Of the patients, 72.5% (*n* = 221) and 72.2% (*n* = 330) had documentation of decision time for emergency cesarean sections in period 1 and 2, respectively (*p* = 0.94). Patients with completed incision time documentation improved from 95.1% to 98.3% (*p* = 0.01) between period 1 and 2. Overall, 540 patients had both decision and incision time completed. Although the number of patients with fully completed decision to incision time interval increased from 69% (*n* = 213) in period 1 to 71.6% (*n* = 327) in period 2, the difference was not statistically significant (*p* = 0.61).

### 3.3. Characteristics of Emergency Cesarean Sections with Complete Decision and Incision Interval

Among the patient records that had complete decision to incision time interval documentation, 36.5%, 21.1%, and 9.8% of the indication were for obstructed labor, cephalo-pelvic disproportion, and antepartum hemorrhage, respectively. Hence, these three main indications contributed to over two thirds of all emergency cesarean sections (Table 1). Malposition, prolonged labor, fetal distress, and ruptured uterus each contributed to less than 6% of all emergency cesarean sections. Around half (52.2%) and about three quarter (73.9%) of the emergency cesarean sections were performed during the day and during weekdays, respectively. In general, more emergency cesarean sections were done in period 2 in comparison to period 1 (Table 1).

### 3.4. Changes in the Decision to Incision Time Interval

From period 1 and period 2, the median decision to incision time interval for emergency cesarean sections decreased from 105 min (IQR 60–122) to 42 min (IQR 25–70) (<0.001). The decision to incision time interval falling within the 30-min benchmark increased from 8.5% (*n* = 18) to 36.75 (*n* = 129) (<0.001). Similarly, there was an increase in emergency cesarean sections that started within 31 min to 75 min (28.6% to 40.7%, *p* < 0.001). Emergency cesarean sections that started after 75 min or later decreased from 62.8% to 22.6% (*p* < 0.001).

Decision to incision interval for obstructed labor, cephalopelvic disproportion, antepartum hemorrhage, and breech and other mal-presentation decreased from between 85–125 min to 40–43 min. Among these same indications, between 35–45% were performed within 30 min. Although not statistically significant, decreases were observed in prolonged labor, fetal distress, and ruptured uterus (Table 2).

Regardless of time of the day or the day of the week, median decision to incision time interval decreased from over 95 min in period 1 to between 35–45 min in period 2 (*p* < 0.001). However, decreases in median time and increases in cesarean section performed with 30 min were higher for cesarean sections performed during the night and over the weekend in periods 1 and 2 (Table 3).

### 3.5. Changes in Decision and Incision Interval and Maternal and Neonatal Outcomes

In 24 months, 19 maternal deaths (11 in period 1 and eight in period 2) were reported. Six neonatal deaths (Five in period 1 and one in period 2) and 16 fresh still births (five in period 1 and 11 in period 2) were recorded. We could not find any statistically significant differences between women and neonates with or without adverse outcomes measured in this study and their decision to incision time interval (Table 4).

## 4. Discussion

As far as we are aware, this is the first study demonstrating challenges with documentation of decision and incision time and the changes in the decision to incision time interval for emergency cesarean sections in Sierra Leone. While documentation in obstetric units in low- and middle-income countries is a known challenge, there is even less information on the routine recording of the decision to incision time interval available in such settings [11,17,22]. In a multi-site study conducted in secondary level facilities with the capacity to conduct cesarean sections in Niger, Guinea, Mali, and Uganda, less than 20% of patients’ charts had decision to incision time interval data [11]. For this study, we reported that 70% of charts had the complete decision time and over 95% had the complete incision time. Although the proportion of charts with both the decision and the incision data completed was about 70%, this is far higher than countries reported in this multi-site study [11].

The study showed a consistent reduction in the decision to incision time interval over the reported 24 months, decreasing from 105 to 42 min. By period 2, 37% of the emergency cesarean sections were performed within 30 min. This suggests that while the median decision to incision time interval for all emergency cesarean sections did not reach the 30-min threshold, considerable heath worker improvements in responding to obstetric emergencies were achieved, especially considering the low and middle income setting of our study.

In this study, the decision to incision time interval decreased significantly regardless of time of the day and the day of the week. However, the decrease was higher for the night and the weekend in comparison to the day, although the decrease was not statistically significant. With evidence that neonatal adverse outcomes commonly occur at night and over the weekend, reduction of the decision to incision time at these times is necessary in a context where this study was done [23,24]. We postulate that the shorter time at night and over the weekend maybe attributed to less work load during these shifts.

Although the audit and feedback program was not implemented to measure the neonatal and maternal outcomes, we attempted to collect selected outcomes in the decision and incision register. Among the few selected outcomes, the study did not find any relationship between the decision to incision time interval with the selected outcomes. It would have been good to explore the relationship between the decision to incision time and immediate outcomes like the Apgar score.

Our results were achieved by building the capacity of the obstetric unit with continuous auditing and feedback of the decision to incision time interval project. These changes were also combined with general health system strengthening initiatives in the unit. We suggest the routine measurements of the decision to incision interval in clinical care in similar settings to KGH. The building of the capacity to improve quality of care and continuous auditing has also been suggested as one of the key enablers to reducing the decision to incision time in high-resources settings [16].

Our study has several limitations. The study focused on the decision to incision time interval as one measure of the quality of the cesarean section. Other measures of the quality of cesarean sections, like decision time to time anesthetist/other care providers arrive in theatre, are equally important and will be addressed in further modifications of this audit and feedback project. This is a single facility study; therefore, findings may not be generalizable to other facilities in Sierra Leone. As the study used routinely collected data, accuracy of cesarean section indications and their recording relied on the skills of doctors and other clinical teams. Our study has missing information, which may affect the validity of our results. However, as an audit study, documenting completeness is essential, as it shows areas that warrant further improvements. We are also aware that our sample size is relatively small. However, the results can inform program managers on how to implement decision to incision quality improvement programs in developing countries.

## 5. Conclusions

The use of continuous audit and feedback quality improvement projects focused on the decision to incision time interval for health system strengthening initiatives in a rural facility resulted in significant reduction in the decision to incision time interval. Additionally, the proportions of emergency cesarean sections performed within 30 min and 75 min increased from 8.5% to 37% and 37% to 77%, respectively. The decision to incision interval decreased across all cesarean indications, as well as the day and time when cesarean sections were performed.

## Figures and Tables

**Table 1:** Characteristics of emergency cesarean section with complete decision to incision interval performed in 24 months (*n* = 540).

	Period 1		Period 2		Total	
	(*n* = 213)	%	(*n* = 327)	%	(*n* = 540)	%
Indications						
Obstructed labor	73	34.3	124	37.9	197	36.5
Cephalopelvic disproportion	52	24.4	62	19.0	114	21.1
Antepartum hemorrhage	25	11.7	28	8.6	53	9.8
Breech in labor & other malposition	12	5.6	22	6.7	34	6.3
Prolonged labor	10	4.7	23	7.0	33	6.1
Fetal distress	12	5.6	20	6.1	32	5.9
Ruptured uterus	12	5.6	11	3.4	23	4.3
Others	17	8.0	37	11.3	54	10.0
Time of day						
Day	128	60.1	154	47.1	282	52.2
Night	85	39.9	173	52.9	258	47.8
Day of week						
Weekday	157	73.7	242	74.0	399	73.9
Weekend	56	26.3	85	26.0	141	26.1
Fetal outcomes						
Alive	191	89.7	302	92.4	493	91.3
Fresh still birth	5	2.4	11	3.4	16	3.0
Neonatal death	5	2.4	1	0.3	6	1.1
Missing outcome	1	0.5	0	0.0	1	0.2
Maternal outcomes						
Alive	201	94.4	319	97.6	520	96.3
Maternal deaths	11	5.2	8	2.5	19	3.5
Missing outcome	1	0.5	0	0.0	1	0.2

Notes: % percentage.

**Table 2:** Incision to decision time interval by indications of emergency cesarean section.

	Average Time (Median, IQR)			within 30 min (n,%)		over 30 min (n,%)			
	Period 1	Period 2	*p*	Period 1	Period 2		Period 1	Period 2	*p*
Obstructed Labor	129 (78–262)	41 (25–65.5)	<0.001	6 (8.2)	45 (36.3)		67 (91.8)	79 (63.7)	<0.001
Cephalopelvic disproportion	107.5 (62.5–192.5)	41.5 (33–65)	<0.001	3 (5.8)	25 (40.3)		49 (94.2)	37 (59.7)	<0.001
Antepartum Hemorrhage	85 (56–225)	42.5 (15–87)	0.002	4 (16)	12 (42.9)		21 (84)	16 (57.1)	0.03
Breech in labor & other malposition	119 (62.5–230)	40.5 (25–79)	0.002	0(0)	8(36.4)		12 (100)	14 (63.4)	<0.001
Prolonged labor	65.0 (25–123)	40.0 (22–60.0)	0.34	3 (30)	8 (34.8)		7 (70)	15 (65.2)	1
Fetal Distress	66.5 (46–208)	44 (35–65)	0.05	1 (8.3)	4 (20)		11 (91.7)	16 (80)	0.63
Ruptured uterus	65 (50–100)	34 (13–72)	0.06	1(8.3)	5(45.4)		11 (91.7)	6 (54.6)	0.07
Others	90 (68–165)	50 (23–112)	<0.001	0(0)	13 (24.1)		17 (100)	24 (64.9)	<0.001

IQR: Interquartile range.

**Table 3:** Decision to incision time interval by time and day of the week.

	Average Time (Median, IQR)			within 30 min (n,%)			over 30 min (n,%)	
	Period 1	Period 2	*p*	Period 1	Period 2	Period 1	Period 2	*p*
Weekend	117.5 (67.5–187.5)	35 (19–58)	<0.001	5 (8.9)	42 (49.4)	51(91.1)	43 (50.6)	<0.001
Weekday	100 (60–225)	45 (25–71)	<0.001	13 (8.3)	78 (32.2)	144 (91.7)	164 (67.8)	<0.001
Night	120 (62–262)	40 (25–61)	<0.001	9 (10.6)	69 (39.9)	76 (89.4)	104 (60.1)	<0.001
Day	95.5 (60–181)	44 (25–75)	<0.001	9 (7.0)	51 (33.1)	119 (93.0)	103 (66.9)	<0.001

IQR: interquartile range.

**Table 4:** Decision to incision time and selected maternal and neonatal outcomes.

Neonatal Outcomes	Fresh Still Births			Neonatal Deaths	
		Period 1	Period 2	Period 1	Period 2
	Median, IQR ^#^	65 (60–65)	42 (25–95)	104 (58–125)	60
	within 30 min ^+^	0 (0)	3 (100)	0 (0)	0(0)
	>30 min ^+^	5(38.5)	8 (61.5)	5 (83.3)	1 (16.7)
Maternal deaths		Period 1	Period 2		
	Median, IQR ^#^	72 (55–122)	60.5 (36–87)		
	within 30 min ^+^	2 (2.3)	2 (1.7)		
	>30 min ^+^	9(8.7)	6(6.3		

^#^ Median decision to incision time interval (minutes). IQR: interquartile range min minutes. ^+^ number (percentage). In all the outcomes, *p*-values are over 0.05 between period 1 and period 2.

## Data Availability

All data used for this study are available from the corresponding author upon request.

## Supplementary Materials

The following are available online at https://www.mdpi.com/article/10.3390/ijerph18168581/s1, Table S1: Partners in Health (PIH) support to obstetric unit of Koidu Government Hospital in 2016–2017, Table S2: Indications of Cesarean section.
