# Supplementary material for: Reducing Decision to Incision Time Interval for Emergency Cesarean Sections: 24 Months’ Experience from Rural Sierra Leone
Source: Int J Environ Res Public Health. 2021 Aug 13;18(16):8581. doi: 10.3390/ijerph18168581 (PMC8392021; doi:10.3390/ijerph18168581)
Supplement: Supplementary file 1 [file ijerph-18-08581-s001.zip › ijerph-1315882-supplementary/supplementary tables/supplementary Table S1.pdf]

Supplementary Table S1. Partners In Health (PIH) support to obstetric unit of Koidu Government Hospital in 2016-2017.

**Comprehensive support provided to KGH by Partners In Health (PIH) in 2017**
1. Strengthened clinical accompaniment, service provision and mentorship in the obstetric unit. PIH supported the district with essential staff including 1 Obstetric-Gynecologist, 1 international Nurse-Midwife, 1 international nurse educator, 1 nurse anesthetist, 3 mid-level providers and 2 nurses. It was essential to add the anesthetist to ensure full availability at night and weekend throughout the year.<br>2. Conducted regular clinical trainings to obstetric staff<br>3. Developed a set of maternity protocols and job aides for standardization and improvement of care in areas where existing ministry of health and sanitation guidelines did not exist<br>4. Strengthened blood bank services and procured equipment, supplies and consumables necessary for blood donation campaigns<br>5. Provided essential infrastructure support, for example ensuring the availability of power supply<br>6. Supported the laboratory and pharmacy systems to ensure drugs and supplies for the maternity ward are available<br>7. Supported the execution of monthly Maternal Death Surveillance and Response meetings and other data review meetings<br>8. Supported roll out of quality improvement initiatives

*These indications are often interrelated, hence they are mixed in our settings.
