# Supplementary material for: Reducing Decision to Incision Time Interval for Emergency Cesarean Sections: 24 Months’ Experience from Rural Sierra Leone
Source: Int J Environ Res Public Health. 2021 Aug 13;18(16):8581. doi: 10.3390/ijerph18168581 (PMC8392021; doi:10.3390/ijerph18168581)
Supplement: Supplementary file 1 [file ijerph-18-08581-s001.zip › ijerph-1315882-supplementary/supplementary tables/supplementary Table S2.pdf]

Supplementary Table S2. Indications of Cesarean section.

Indication	Description
Prolonged labor*	Labor lasting more than 20 hours for primigravida and more than 14 hours for multigravida.
Cephalopelvic disproportion*	Failure of the baby's head to descend due to size mismatch between the mother's pelvis and the baby's head despite strong contractions
Obstructed Labor*	Failure of fetal presenting part to descend through pelvic canal as a result of mechanical obstruction.
Ruptured uterus	Mainly confirmed during cesarean section. The uterine wall tears apart during childbirth resulting in fetal distress and maternal hemorrhage
Antepartum hemorrhage	Bleeding from or into genital tract after 28 weeks pregnancy and prior to the birth of the baby
Umbilical cord prolapse	Umbilical cord comes out of the uterus before presenting part of fetus
Fetal distress	When the fetus shows signs that they may not be well. This is suggested by decreased fetal movement, meconium stained liquor, fetal heart less than 100 bits/minute or fetal heart more than 160 bits/minute
Breech in labor	A fetus position in longitudinal lie with buttocks closest to the cervix during child birth
Malposition	Abnormal position of the vertex during child birth
Other malpresentation	Any fetal presentation other than vertex presentation and breech engaging the mother's pelvis
Hypertensive disorder in pregnancy	Any cesarean section done as a result of pre-ecclampsia or ecclampsia
Failed vacuum assisted delivery	In this case, the pregnant woman meets the indications for vacuum assisted delivery and the procedure has failed. Decision of failed vacuum extraction is made if one of the following requirements are reached 1) Fetal head does not advance with each pull 2) Fetus is undelivered after three pulls with no descent, or after 20-30 minutes 3) Cup slips off the head twice at the proper direction of pull with a maximum negative pressure.
Labor dystocia	Abnormally slow dilatation of cervix when the woman is in active phase of labor (Dilatation less than 1/2 cm per hour for 4 hours)
Others	Due to small numbers, these indications were included in the "others" section of the manuscripts. They included failure of induction, retained second twin, post term, poor obstetric history, previous cesarean section, and multiple gestation.

*These indications are often interrelated, hence they are mixed in our settings.
